# Analysis of self-renewing and differentiation-related microRNAs and transcription factors in multilineage mouse hematopoietic stem/progenitor cells induced by 1,4-benzoquinone

**DOI:** 10.7717/peerj.15608

**Published:** 2023-07-10

**Authors:** Ramya Dewi, Nur Afizah Yusoff, Siti Razila Abdul Razak, Zariyantey Abd Hamid

**Affiliations:** 1 Biomedical Science Programme and Centre of Diagnostic, Therapeutic and Investigative Science, Faculty of Health Sciences, Universiti Kebangsaan Malaysia, Jalan Raja Muda Abdul Aziz, Kuala Lumpur, Malaysia; 2 Oncological and Radiological Sciences Cluster, Advanced Medical and Dental Institute, Universiti Sains Malaysia, Bertam, Kepala Batas, Pulau Pinang, Malaysia

**Keywords:** Benzene, 1,4-Benzoquinone, Hematopoietic stem/progenitor cells, Lineages, Epigenetic, MicroRNA, Transcription factors

## Abstract

**Background:**

HSPCs are targets for benzene-induced hematotoxicity and leukemogenesis. However, benzene toxicity targeting microRNAs (miRNAs) and transcription factors (TF) that are involve in regulating self-renewing and differentiation of HSPCs comprising of different hematopoietic lineages remains poorly understood. In this study, the effect of a benzene metabolite, 1,4-benzoquinone (1,4-BQ) exposure, in HSPCs focusing on the self-renewing (miRNAs: miR-196b and miR-29a; TF: HoxB4, Bmi-1) and differentiation (miRNAs: miR-181a, TF: GATA3) pathways were investigated.

**Methods:**

Freshly isolated mouse BM cells were initially exposed to 1,4-BQ at 1.25 to 5 µM for 24 h, followed by miRNAs and TF studies in BM cells. Then, the miRNAs expression was further evaluated in HSPCs of different lineages comprised of myeloid, erythroid and pre-B lymphoid progenitors following 7–14 days of colony forming unit (CFU) assay.

**Results:**

Exposure to 1,4-BQ in BM cells significantly (*p* < 0.05) reduced the miR-196b (2.5 and 5 µM), miR-181a (1.25, 2.5 and 5 µM) and miR-29a (1.25 µM) along with upregulation of miR-29a at 2.5 µM. Meanwhile, 1,4-BQ exposure in HSPCs significantly increased the miR-196b expression level (*p* < 0.05) only in myeloid and pre-B lymphoid progenitors at 2.5 and 5 µM. Significant (*p* < 0.05) reduction in expression of miR-181a in myeloid (1.25 µM), erythroid (5 µM) progenitors along with miR-29a in myeloid (1.25 µM) and pre-B lymphoid (5 µM) progenitors were noted following exposure to 1,4-BQ. Meanwhile, increased expression of miR-181a was observed in pre-B lymphoid progenitor upon exposure to 1,4-BQ, but only at 5 µM. As for TF studies, expression of HoxB4 protein was significantly increased (*p* < 0.05) at all 1,4-BQ concentrations as compared to Bmi-1 and GATA3, which were significantly (*p* < 0.05) elevated starting at 2.5 µM of 1,4-BQ.

**Conclusion:**

1,4-BQ induces aberration of miRNAs and transcription factors protein expression that are involved in regulating self-renewing and differentiation pathways of HSPCs. Moreover, epigenetic toxicity as evidenced from the miRNAs expression was found to be mediated by a lineage-driven mechanism. The role of cell lineage in governing the toxicity of 1,4-BQ in HSPCs lineages deserves further investigation.

## Introduction

Benzene is a harmful environmental pollutant that is present in vehicle exhaust, cigarette smoke and is used in many industries (Vitale & Gutovitz, 2020). Exposure to benzene has been associated with increased risk of hematological dysfunctions and malignancies, such as aplastic anemia, myelodysplastic syndrome and acute myeloid leukemia, indicating the role of the hematopoietic system as a chief target of benzene’s toxic effects (McHale et al., 2011; Qian et al., 2019). It is believed that benzene can cause hematotoxicity through numerous mechanisms, including oxidative damage, gene mutation and epigenetic changes (Dewi et al., 2019; Kasemy et al., 2019; Wang et al., 2012a; Wang et al., 2012b). Although extensive studies on potential health hazards associated with benzene exposure have been done, the molecular mechanisms mediating benzene hematotoxicity remains to be elucidated.

Hematopoiesis is a blood-forming process that is highly regulated and maintained by complex molecular factors that regulate the formation of all mature blood cell types, particularly *via* self-renewing and differentiation properties of HSPCs (Greim et al., 2014). Thus, alterations in hemostasis between self-renewing and differentiation properties of HSPCs could lead to the accumulation of abnormal stem cells and/or progenitors and subsequent development of hematological disorders and malignancies (Lazare et al., 2014). Previous studies have reported some evidence of benzene toxicity affecting HSPCs self-renewing and differentiation pathways that lead to the clonal expansion of leukemic stem cells (LSCs) (Greim et al., 2014; Synder, 2012). Moreover, a study conducted by (Man et al., 2018) have reported that genes involved in HSPCs self-renewing, cell cycle, differentiation and apoptosis pathways were found to be significantly reduced upon benzene exposure and may participate in benzene induced leukemogenicity. Despite these reported findings, not much is known on the effect of benzene exposure and/or its metabolite on hematopoietic stem cell (HSCs) niche comprising of different lineages along with its underlying mechanism targeting self-renewing and differentiation pathways.

MicroRNAs (miRNAs) have been shown to cooperate with transcription factors to regulate all aspects of hematopoiesis (Dewi et al., 2019). MiRNAs act as a key regulator in hematopoiesis, including in cell transitions, lineage selection, maturation and terminal differentiation, thus illuminating essential roles for miRNAs in controlling the quiescence state and self-renewing of HSCs as well as proliferation and differentiation of line-age-specific progenitors during erythropoiesis, myelopoiesis and lymphopoiesis (Kim, Civin & Kingsbury, 2019). MiRNAs function as downstream effectors of hematopoietic transcription factors and as upstream regulators to control transcription factors in which different MiRNAs families are shown to oppose one another in the regulation of HSCs self-renewing and differentiation (Palma et al., 2014). Since miRNAs are important in hematopoiesis, it is no surprise that dysregulation of miRNAs expression has been increasingly implicated in the development and pathogenesis of hematologic malignancies (Lazare et al., 2014). Recent discoveries implicated the dysregulation of miRNAs expression in the initiation and progression of leukemia, as they can act as oncogenes (oncomiRs) and tumor suppressors (Ogawa, 2014). MiRNAs have been observed to contribute to myelodysplastic syndromes (MDSs) by increasing the proliferation and self-renewing of human and mouse megakaryocytic progenitors and megakaryocytic/erythroid progenitors (Bai et al., 2014). Furthermore, aberrant miRNA expression was also reported in benzene-induced hematotoxicity. Aberrant miRNA profiles were first reported in peripheral blood mononuclear cells of chronic benzene-exposed patients (Liang et al., 2018). In the following year, an aberrant miRNA expression profile in benzene-induced HSPCs in murine bone marrow was also reported, signifying the importance of studying molecular mechanisms underlying benzene hematotoxicity associated with miRNAs at HPSCs. Furthermore, a previous study reported that benzene inhalation mediated erythroid toxicity in mice *via* the downregulation of miRNAs involved in erythroid cell differentiation (Chow et al., 2015). This finding provides new insight into the mechanism underlying benzene hematotoxicity mediated by miRNAs at the lineage specific level of HSPCs.

Recently, our group has demonstrated a lineage-dependent response as emerging evidence linking HSPCs of different lineages toward benzene toxicity (Chow et al., 2015). These studies showed that the clonogenicity of myeloid progenitors was selectively inhibited following 1,4-BQ exposure, suggesting the role of lineage-specificity in governing differential cytotoxicity effects of benzene in targeting the HSC niche. In addition, a previous study found that 1,4-BQ exposure at non-cytotoxic concentrations modulates the fate of HSPCs by altering the genes regulating the self-renewal and differentiation pathway (Chow et al., 2018). Thus, in the present study, we evaluated the toxicity effect of 1,4-BQ on protein expression of three transcription factors (HoxB4, Bmi-1 and GATA 3) to investigate whether the protein expression is influenced by the changes in the gene expression level as any improper regulation in the activity of these complex molecular networks may interfere in the HSPCs fate, resulting in cellular transformation and subsequent malignancies (Wei et al., 2015).

It is well understood that the acquisition of self-renewing by LSCs is a typical component for the malignant properties of leukemias. Moreover, dysregulation of miRNAs expression is often associated with hematopoietic malignancies involving the self-renewing and differentiation pathways. For example, miR-196b itself has vital roles in maintaining the undifferentiated state of primitive HSCs by repressing genes involved in differentiation while miR-181a and miR-29 play multiple roles in the progression of neoplasia and leukemia. Hence, to clearly demonstrate the effects of such exposure established in an *in vitro* 1,4-BQ toxicity model, we studied the alteration in miRNAs profiles (miR-196b, miR-181a and miR-29a) as well as transcription factors (HoxB4, GATA 3 and Bmi-1) involved in regulating self-renewing and differentiation properties of HSPCs with targeted effect on different HSPCs lineages comprised of myeloid, erythroid and pre-B lymphoid progenitors. Overall, this study is believed to elucidate a novel mechanism of benzene-induced hematotoxicity and leukemogenicity targeting the HSPCs niche *via* a lineage-directed strategy.

## Materials and Methods

### Experimental design

**Figure 1:**
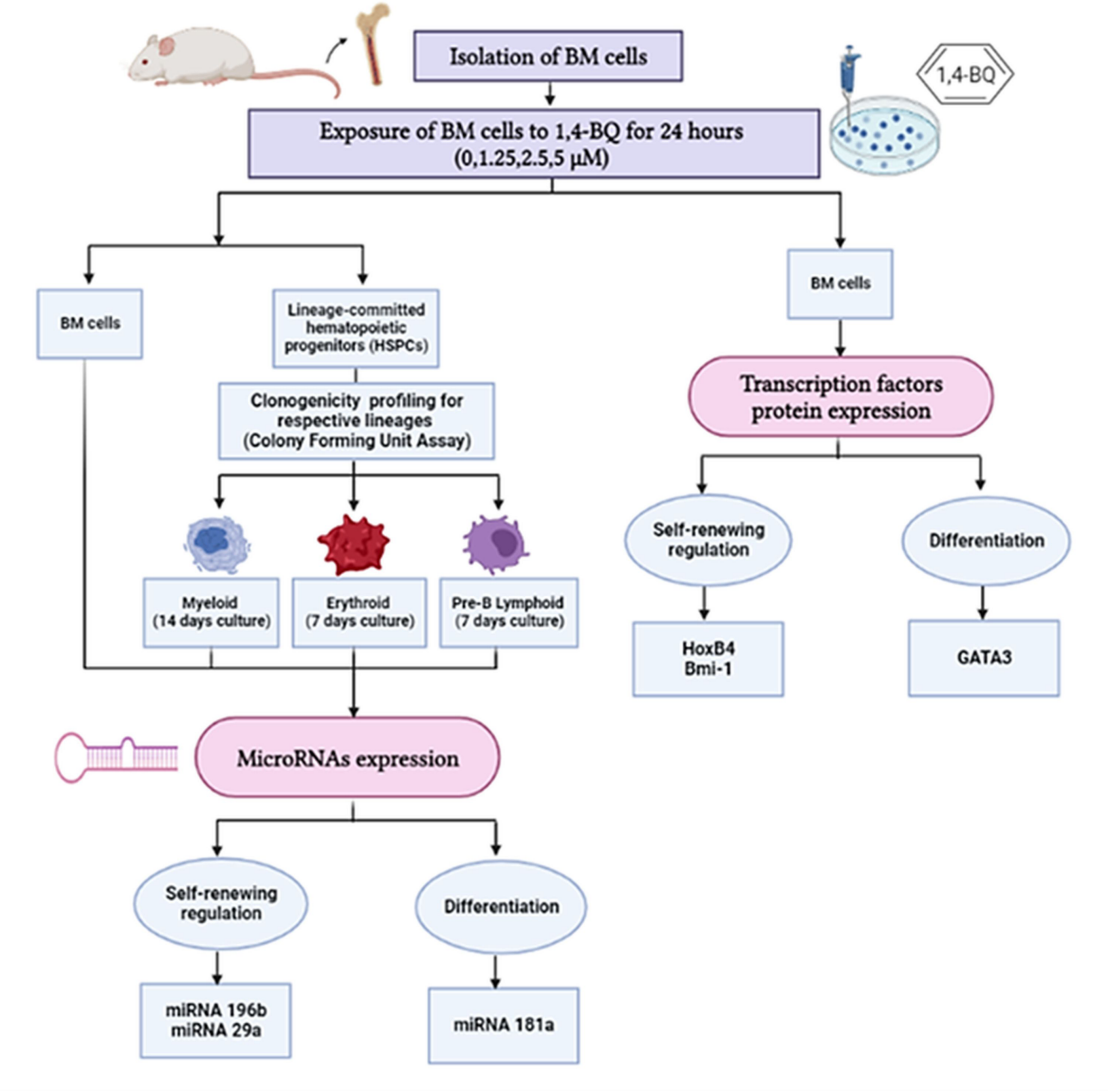
Research design demonstrating the experimental workflow. Research design demonstrating the experimental workflow on the effects of 1,4-BQ exposure for 24 h targeting the BM cells and HSPCs of different lineages comprise of myeloid, erythroid and pre-B lymphoid progenitors.

In this study, we investigated protein expression of transcription factors in BM cells and the miRNAs expression in both BM cells and HSPCs comprised of myeloid, erythroid and pre-B lymphoid lineages. The research design for the current study is shown in Fig. 1.

### Isolation of mouse bone marrow cells and culture condition

The procedures that involve the use of mice (*Mus musculus musculus*) were approved by the Animal Ethics Committee of the Universiti Kebangsaan Malaysia (UKMAEC, Kuala Lumpur, Malaysia) with the approval number FSK/2016/ZARIYANTEY/23-NOV/814-NOV-2016-JULY-2019-AR-CAT2. Male imprinting control region-strained (ICR) mice were purchased from the Laboratory Animal Resource Unit, Faculty of Medicine, Universiti Kebangsaan Malaysia, Kuala Lumpur, Malaysia. Anesthesia was not used in this study to avoid sample contamination with chemical and toxicity that could affect the quality of bone marrow cells prior to downstream analysis and subsequent scientific outcomes. Thus, the mice were sacrificed *via* cervical dislocation following method as described (Hickman & Johnson, 2011). Briefly, the cervical dislocation was performed by placing the finger or an instrument such as scissors behind the base of the skull and quickly pushing downward and forward the head while pulling the tail firmly with the hand to achieve rapid separation of cervical tissues. The ICR mice were used in the current study to maintain a similar phenotype of laboratory animals being used for the isolation of BM cells and HSPCs as reported in previous studies by our group (Chow et al., 2015; Chow et al., 2018; Dewi et al., 2019). BM cells were obtained through femur and tibia flushing from 10-week-old adult male mice as previously described (Dewi et al., 2019). Collected BM cells were filtered through a 40 µM nylon mesh (BD Biosciences, San Jose, CA, USA) and suspended in complete cell culture medium consisting of DMEM (Dulbecco’s Modified Eagle Medium) supplemented with 10% fetal bovine serum (FBS; JRS Scientific Inc., Woodland, Canada), 1% penicillin/streptomycin (PAA Laboratories GmbH, Pasching, Australia), 100 ng/mL stem cells factor (SCF), 10 ng/mL interleukin-6 (IL-6) and 5 ng/mL interleukin-3 (IL-3) (Miltenyi Biotec, Bergisch Gladbach, Germany). Cells were then maintained overnight in a humidified incubator at 37 °C and 5% CO_2_.

### Exposure of mouse bone marrow cells to 1,4-BQ

A stock solution of 50 mM 1,4-BQ (CAS No: 106-51-4 and ≥ 98% purity) obtained from Sigma-Aldrich Co. (St. Louis, MO, USA) was freshly prepared in a phosphate buffer solution. Mouse BM cells (1 × 10^6^ cells/mL) were exposed for 24 h at 37 °C and 5% CO_2_ to non-cytotoxic concentrations of 1,4-BQ (1.25 and 2.5 µM) and a cytotoxic concentration (IC_10_ = 5 µM) as previously determined by Liang et al. (2018).

### Colony forming unit for myeloid, erythroid and pre-B lymphoid progenitors

Briefly, colony forming unit (CFU) assay is an in-vitro assay that enables the study of hematopoietic stem / progenitor cells (HSPCs) function through their potency to proliferate and differentiate into myeloid, erythroid and lymphoid lineages. For this purpose, a fixed number of input HSPCs are cultured on a semisolid medium containing growth factors which allow the measurement of their proliferative and differentiation potency through the colony-forming ability. The morphology and number of colonies can be analyzed and the colonies can be harvested for downstream analysis. In this study, three different methylcellulose culture media with specialized formulations of growth factors acquired from StemCell Technologies (Vancouver, British Columbia, Canada) were used in the CFU assays to support the growth of lineage-committed hematopoietic progenitors. The three progenitors were myeloid (colony-forming unit-granulocyte macrophage (CFU-GM), colony-forming unit macrophage (CFU-M) and colony-forming unit granulocyte (CFU-G)(MethoCult media #03534)), erythroid (colony-forming unit-erythroid (CFU-E) and mature burst-forming unit-erythroid (BFU-E) (MethoCult media #03334)) as well as pre-B lymphoid (colony-forming unit-Pre-B (CFU-Pre-B) (MethoCult #03630)). The assay was performed as previously described (Wei et al., 2015).

In brief, following 24 h of 1,4-BQ exposure, BM cells were harvested from the culture by centrifugation at 2500 rpm for 7 min. The cells were resuspended in fresh medium followed by cell count for determination of cell viability. The viable cells were then prepared in 100 µL of medium containing 1 × 10^5^, 2 × 10^4^ and 5 × 10^4^ cells for preparation of myeloid, erythroid and pre-B lymphoid progenitors CFU, respectively. Then, the cells were plated in 1 mL of methylcellulose culture medium in a 35 mm plate. The CFU cultures were incubated at 37 °C with 5% CO_2_ for 7 days (CFU-E, mature BFU-E and CFU-pre-B lymphoid) and 14 days (CFU-GM, CFU-G, and CFU-M). Formed colonies from respective progenitors were harvested and a single cell suspension was collected *via* resuspension of the semi-solid CFU medium in DMEM, followed by centrifugation at 2500 rpm for 7 min prior to downstream analysis.

### RNA isolation and quantitative RT-PCR

In this study, we investigated the miRNAs expression using quantitative RT-PCR (qRT-PCR) for miR-196b, miR-181a and miR-29a in BM cells following 24 h exposure to 1,4-BQ and subsequent miRNAs expression analysis in hematopoietic progenitors after CFU cultures for erythroid and pre-B lymphoid (7 days) and myeloid (14 days). Briefly, after 24 h of 1,4-BQ exposure, BM cells were harvested for two downstream analysis as follows: (i) miRNAs isolation and expression analysis (ii) CFU assay for subsequent miRNAs isolation and expression study in myeloid, erythroid and pre-B lymphoid progenitors.

qRT-PCR MiRNeasy Mini Kit (217004 miRNeasy Mini Kit) was used for the extraction of total RNA according to the manufacturer’s instructions from harvested BM cells that were collected *via* centrifugation at 2500 rpm for 7 min. Meanwhile, for lineage-committed hematopoietic progenitors, the total RNA extraction was performed after the CFU colonies were harvested and centrifuged at 2500 rpm for 7 min after multiple resuspensions in DMEM to isolate single cell suspensions. Then, RNA purity was evaluated by a NanoDrop ND-1000 spectrophotometer.

Reverse transcription (RT) reaction was performed using the TaqMan MicroRNA Reverse Transcription Kit (Applied Biosystems, Thermo Fisher Scientific, Waltham, MA, USA) and miRNA-specific primers according to the manufacturer’s protocol. Quantitative RT-PCR analysis of miRNA expression was carried out using the following Taqman probes: mmu-miR-196b-3p, TM: 002215; mmu-miR-181a-3p. TM: 000480; mmu-miR-29a-3p, TM: 002112 (Applied Biosystems Foster, CA, USA). Briefly, the total RNA was transcribed into cDNA which was used as the template for miRNA qRT-PCR analysis. Then, the reverse transcription reaction was conducted following manufacturer recommendations. The mixture was then transferred to the thermal cycler and reverse transcription was performed at 16 °C for 30 min, 42 °C for 30 min, 85 °C for 5 min and at 4 °C holding period. The real-time PCR protocols included 95 °C for 10 min; 40 cycles of 95 °C for 15 s, 60 °C for 60 s; and a 4 °C holding period. All samples were analyzed in triplicate and the relative expression levels of miRNAs were normalized against the U6 housekeeping miRNAs gene (TM: 001973; Applied Biosystems). The 2 − Δ ΔCT method was used to determine the relative expression compared to the control. The expression data for miRNAs were acquired and analyzed with the StepOnePlusTM real-time PCR systems and StepOne Software v2.2.2 (Applied Biosystems, Foster, CA, USA).

### ELISA for transcription factors protein analysis

The expression of HoxB4, Bmi-1 and GATA3 proteins were determined using ELISA protocol as recommended by the supplier (Bioassay Technology Laboratory and Elabscience, China). Prior to downstream ELISA analysis, antibody specific for HoxB4 (#SEH890Mu; Cloud-Clone Corporation, Houston, TX, USA), Bmi-1 (#E2018Mo; Bioassay Technology Laboratory, China) and GATA3 (#ELSE-EL-M0545; Elabscience, China) has been pre-coated onto a microplate. Briefly, following the 24 h 1,4-BQ exposure, protein lysate from BM cells were harvested through centrifugation of BM cells at 2500×g for 5 min and the cell pellets were rinsed with cold PBS. This step was repeated twice to obtain cell pellets free of medium residues. Then, 1 mL of RIPA buffer solution, 10 µL protease enzyme inhibitor PMSF (phenylmethylsulphonyl fluoride) and 10 uL phosphotyrosyl phosphatases (PTPs) inhibitor Na_3_VO_4_ (sodium orthovanadate) were added to the obtained cell pellet and continuously shaken for 15 min on ice. Then the solution was centrifuged at 14,000 x g for 15 min to obtain the supernatant. The protein lysate was stored at −80 °C prior to analysis. Then, ELISA assay was performed using specific procedures as recommended by the supplier (Cloud-Clone Corporation, Houston, TX, USA; Bioassay Technology Laboratory and Elabscience, China) for respective HoxB4, Bmi-1 and GATA3 proteins and the expression levels were measured spectrophotometrically of 450 nm. Normalization of input for ELISA analysis was achieved using standardized amount of protein concentration for every experimental group at 2 mg/ml using bicinchoninic acid (BCA) protein quantification method. Then, data were analyzed through statistical comparison of 1,4-BQ treated groups to that of the negative control (untreated group).

### Statistical analysis

Data are shown as mean ± SEM. Results were first evaluated for homogeneity of variance. One-way ANOVA was performed and when the variances were not equal, the non-parametric Mann–Whitney test was employed. We considered *p* < 0.05 to be significant. All results were analyzed using the SPSS version 23.0 software (SPSS, IBM Corporation, Armonk, New York, USA).

## Results

### miR-196b expression in 1,4-BQ-exposed BM cells and differential lineages of hematopoietic progenitors

**Figure 2:**
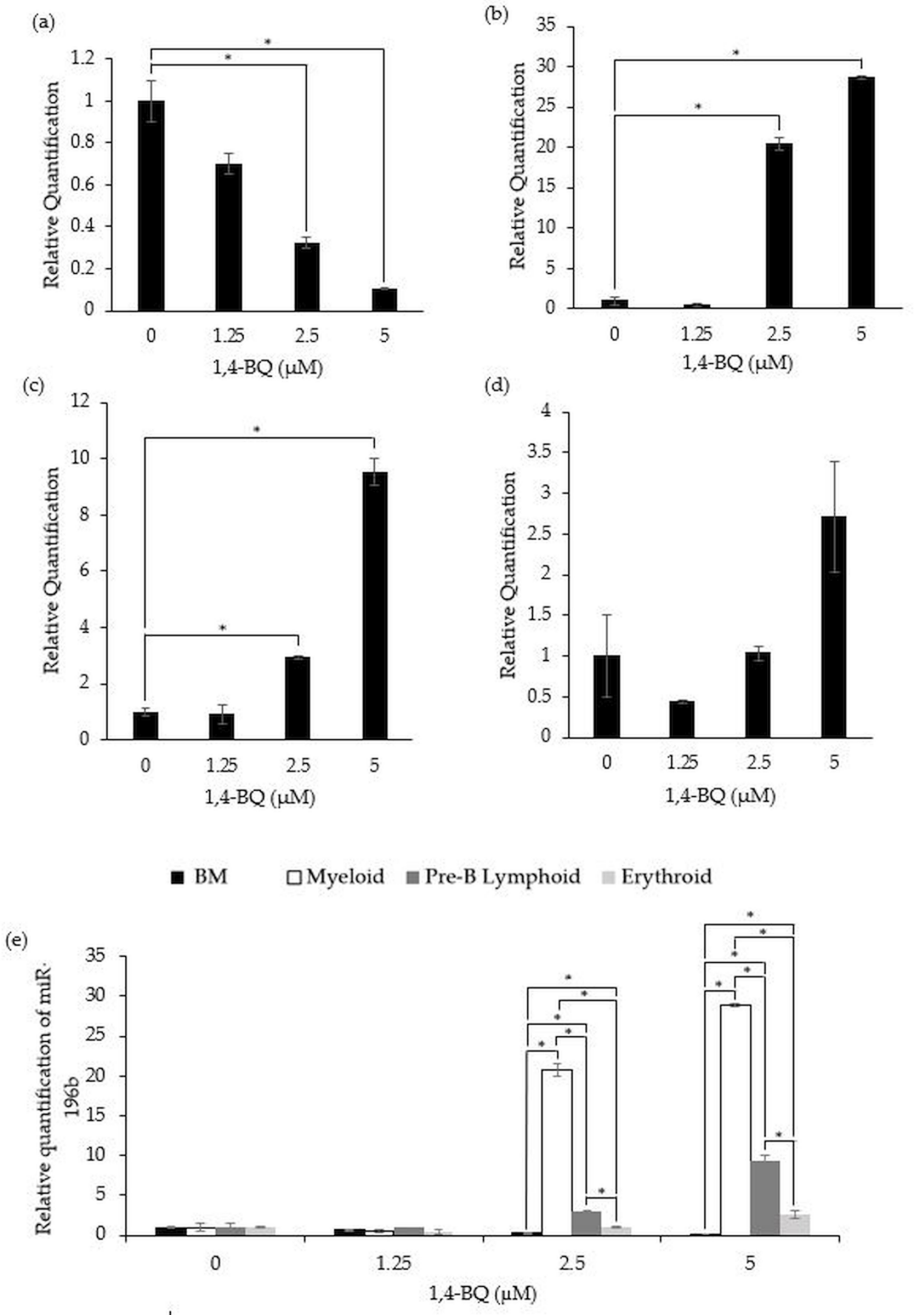
Effect of 1,4-BQ exposure on the level and comparative miR-196b expression in respective groups. Effect of 1,4-BQ exposure on the level of miR-196b expression BM cells (A), myeloid progenitor (B), pre-B lymphoid progenitor (C), erythroid progenitor (D) and comparative miR-196b expression between BM cells and differential lineages of hematopoietic progenitors (E). Data are presented as the mean ± SEM (*n* = 3). * *p* < 0.05 for comparison between the indicated groups.

Figure 2 shows the level of miR-196b expression following treatment with 1,4-BQ on BM cells and hematopoietic progenitors of myeloid, pre-B lymphoid and erythroid lineages. Notably, exposure to 1,4-BQ induced downregulation on the level of miR-196b expression in a dose-dependent manner for BM cells of which significant (*p* < 0.05) reduction were observed following 1,4-BQ exposure at 2.5 (0.33 ± 0.03) and 5 µM (0.10 ± 0.01) as compared to the untreated group (1.00 ± 0.10) (Fig. 2A). Meanwhile, the level of miR-196b expression was upregulated significantly (*p* < 0.05) in the myeloid (20.8 ± 0.78) and (28.89 ± 0.18) as well as pre-B lymphoid progenitors (3.02 ± 0.09) and (9.38 ± 0.67) upon 1,4-BQ exposure at 2.5 and 5 µM as shown in (Figs. 2B and 2C). In contrast, no significant (*p* > 0.05) effect on miR-196b expression was noted in the erythroid progenitor following 1,4-BQ exposure (Fig. 2D). Meanwhile, comparative miRNAs expression between the BM cells and differential lineages of hematopoietic progenitors indicates that myeloid, pre-B lymphoid and erythroid progenitors showed significantly higher (*p* < 0.05) miR-196b expression compared to BM cells at the respective 2.5 µM (20.8 ± 0.78, 3.02 ± 0.09, 1.027 ± 0.08) and 5 µM (28.89 ± 0.18, 9.38 ± 0.67, 2.65 ± 0.05) of 1,4-BQ concentrations (Fig. 2E).

### miR-181a expression in 1,4-BQ-exposed BM cells and differential lineages of hematopoietic progenitors

**Figure 3:**
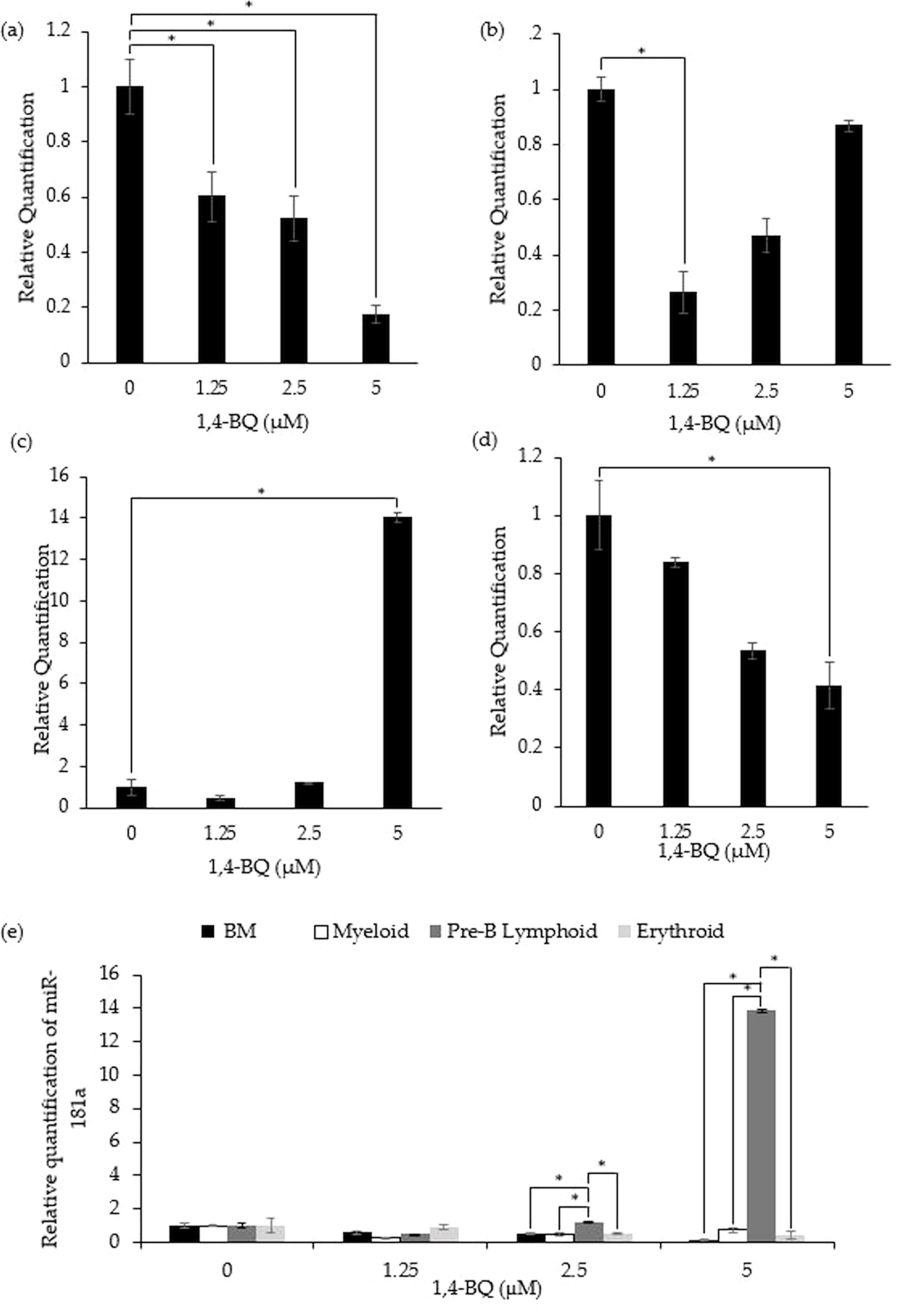
Effect of 1,4-BQ exposure on the level and comparative miR-181a expression in respective groups. Effect of 1,4-BQ exposure on the level of miR-181a expression in BM cells (A), myeloid progenitor (B), pre-B lymphoid progenitor (C), erythroid progenitor (D) and comparative miR-181a expression between BM cells and differential lineages of hematopoietic progenitors (E). Data are presented as the mean ± SEM (*n* = 3). * *p* < 0.05 for comparison between the indicated groups.

Figure 3 shows the level of miR-181a expression after treatment with 1,4-BQ on BM cells and hematopoietic progenitor of myeloid, pre-B lymphoid and erythroid lineages. Notably, exposure to 1,4-BQ induced significant (*p* < 0.05) downregulation on the level of miR-181a expression in BM cells at all concentrations such as 1.25 µM (0.6 ± 0.09), 2.5 µM (0.53 ± 0.08) and 5 µM (0.17 ± 0.03) (Fig. 3A). Significant downregulation (*p* < 0.05) of miR-181a expression was also observed in myeloid and erythroid progenitors only at 1.25 µM (0.28 ± 0.02) and 5 µM (0.41 ± 0.08) 1,4-BQ, respectively (Figs. 3B and 3D). On the contrary, the level of miR-181a expression was significantly upregulated (*p* < 0.05) upon 1,4-BQ exposure at 5 µM (13.84 ± 0.22) in the pre-B lymphoid progenitor (Fig. 3C). Then, when the miRNAs expression was compared between the BM cells and progenitors, it was notable that the pre-B lymphoid progenitor showed significantly higher (*p* < 0.05) miR-181a expressions as compared to BM cells, myeloid and erythroid progenitors at 2.5 µM and 5 µM of 1,4-BQ exposures (Fig. 3E).

### miR-29a expression in 1,4-BQ-exposed BM cells and differential lineages of hematopoietic progenitors

**Figure 4:**
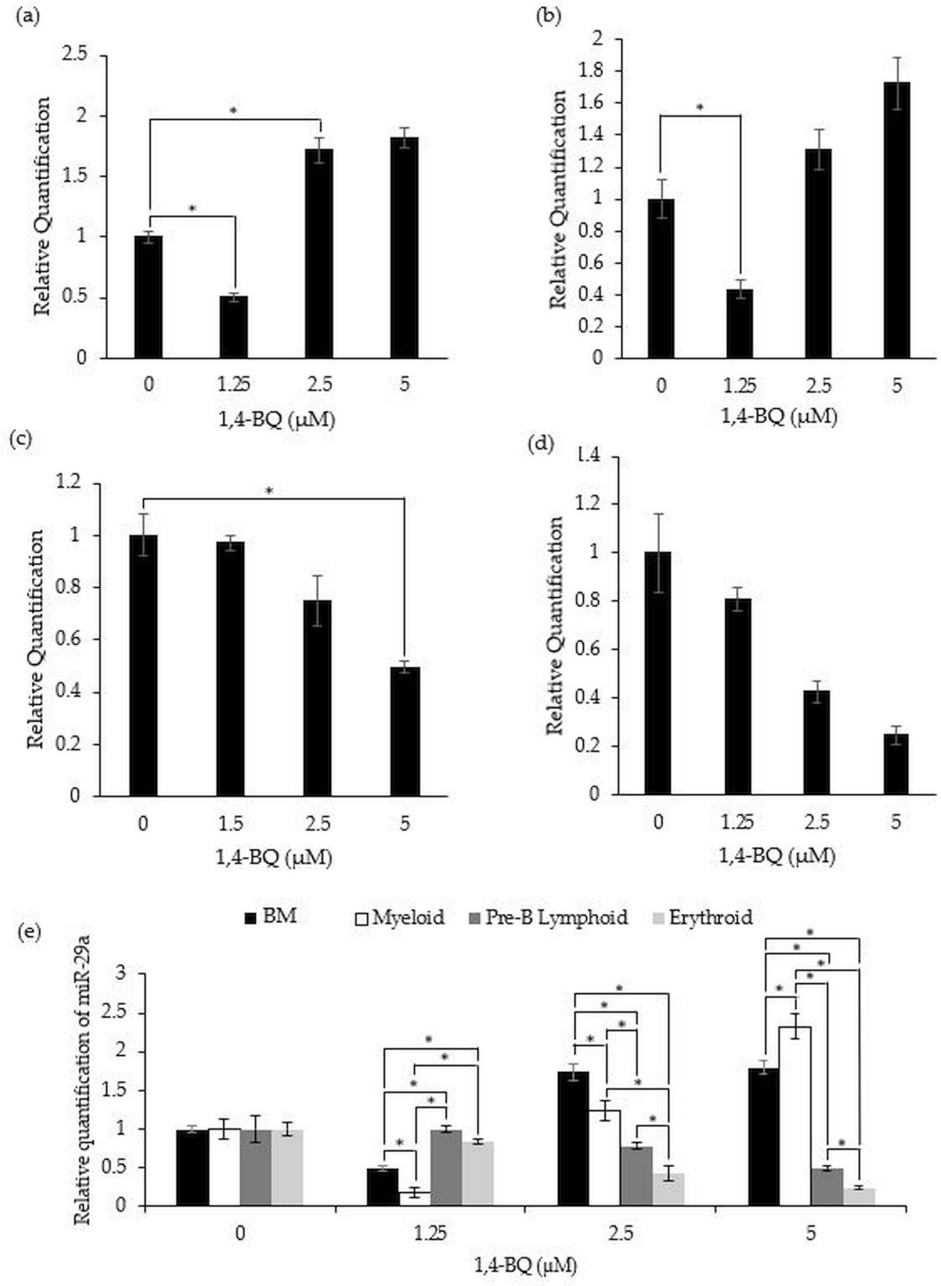
Effect of 1,4-BQ exposure on the level and comparative miR-29a expression in respective groups. Effect of 1,4-BQ exposure on the level of miR-29a expression in BM cells (A), myeloid progenitor (B), pre-B lymphoid progenitor (C), erythroid progenitor (D) and comparative miR-29a expression between BM cells and differential lineages of hematopoietic progenitors (E). Data are presented as the mean ± SEM (*n* = 3). * *p* < 0.05 for comparison between the indicated groups.

Figure 4 shows the level of miR-29a expression after treatment with 1,4-BQ on BM cells and progenitors of myeloid, pre-B lymphoid and erythroid lineages. Notably, a significant downregulation (*p* < 0.05) on the level of miR-29a expression was observed in BM cells (0.50 ± 0.03) and myeloid progenitor (0.18 ± 0.06) (Figs. 4A–4B) at 1.25 µM along as well as in the pre-B lymphoid progenitor (Fig. 4C) at 5 µM (0.49 ± 0.02) with a significant upregulation (*p* < 0.05) at 2.5 µM (1.74 ± 0.11) for BM cells upon 1,4-BQ exposure. Meanwhile, no significant effect following 1,4-BQ exposure on the miR-29a expression was evidenced in the erythroid progenitor (Fig. 4D). Next, across groups comparison between the BM cells and progenitors, it shows that the significant differences (*p* < 0.05) in miR-29a expression were noted in hematopoietic progenitors compared to BM cells, although the trends were inconsistent throughout the 1,4-BQ concentrations (Fig. 4E).

### Transcription factors protein expression in 1,4-BQ-exposed BM cells

**Figure 5:**
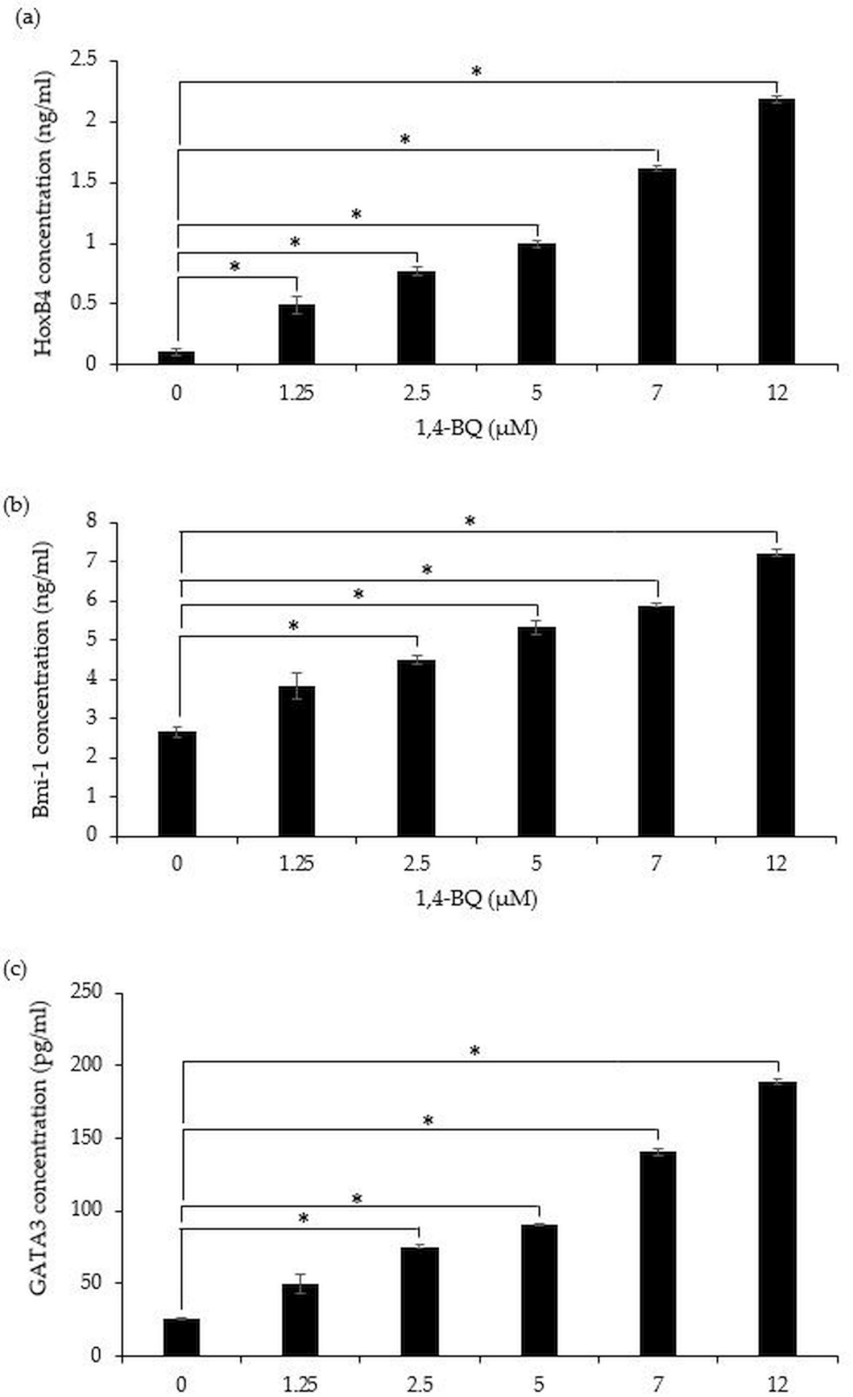
The transcription factors protein expression in 1,4-BQ-exposed BM cells. The transcription factors protein expression in (A) HoxB4, (B) Bmi-1 and (C) GATA3 in mouse BM cells after 1,4-BQ treatment. Data are presented as the mean ± SEM (*n* = 3). * = *p* < 0.05 compared with the untreated group.

A significant (*p* < 0.05) upregulation of HoxB4 protein was evidenced starting at 1.25 µM (0.49 ± 0.07 ng/ml), 2.5 µM (0.77 ± 0.04 ng/ml), 5 µM (0.99 ± 0.03 ng/ml), 7 µM (1.62 ± 0.02 ng/ml) and 12 µM (2.18 ± 0.02 ng/ml) upon 1,4-BQ exposure (Fig. 5A). Meanwhile for Bmi-1 and GATA3 significantly upregulated (*p* < 0.05) were notable at 2.5 µM (4.48 ± 0.11 ng/ml), 5 µM (5.32 ± 0.16 ng/ml), 7 µM (5.89 ± 0.05 ng/ml) and 12 µM (7.21 ± 0.09 ng/ml) as well as 2.5 µM (75.55 ± 0.79 pg/ml), 5 µM (90.74 ± 1.01 pg/ml), 7 µM (140.58 ± 2.81 pg/ml) and 12 µM (189.03 ± 1.69 pg/ml) (Figs. 5B–5C) upon 1,4-BQ exposure as compared to the untreated group.

## Discussion

Benzene is well-known for its ability to induce hematotoxicity and leukemogenicity. Indeed, it has been proven by previous studies that benzene acts *via* multiple modes of action in promoting leukemogenesis involving both genetics and epigenetics mechanisms. The emerging role of miRNAs and transcription factors have been widely reported in previous studies, which indicate their vital roles in the physiological and pathological processes. Additionally, the role of miRNAs in benzene-induced hematotoxicity has also been described previously (Bai et al., 2014; Wei et al., 2015). However, despite the reported findings, the mechanism linking miRNAs and transcription factors in benzene-induced hematotoxicity and leukemogenicity targeting HSPCs niche remains unclear. Thus, in the present study, we investigated the effect of a benzene metabolite exposure, namely 1,4-BQ on the expression of miRNAs (miR -196b, -181a and -29a) and transcription factors (HoxB4, Bmi-1 and GATA3) that are vital in the regulation of self-renewing and differentiation potencies of HSPCs during hematopoiesis. Disturbances to these processes can induce unlimited HSPCs proliferation along with blocked differentiation leading to the accumulation of immature cell populations known as LSCs. Thus, we believe that a study focusing on the miRNAs and transcription factors regulating self-renewing and differentiation pathways in controlling HSPCs fate could uncover potential mechanisms underlying benzene-induced hematotoxicity and leukemogenicity targeting the HSPCs niche. Moreover, to the best of our knowledge, our study is the first to report such a mechanism that is mediated by benzene targeting the HSPCs niche for respective progenitors of erythroid, pre-B lymphoid and myeloid lineages.

Overall, the current study discovered that 1,4-BQ exposure induced differential effects in mediating hematopoietic miRNAs expression between the BM cells in comparison to erythroid, myeloid and pre-B lymphoid progenitors. For miR-196b, its expression was affected following 1,4-BQ exposure and the effect was on the cell types/lineages. The observed effect was based on the significant downregulation of miR-196b expression in BM cells at selected concentrations in comparison to myeloid and pre-B lymphoid progenitors of which expression is upregulated following 1,4-BQ exposure. Moreover, the level of miR-196b expression in the erythroid progenitor was unaffected following 1,4-BQ exposure. Furthermore, comparative assessment indicated that the miR-196b expression was higher in hematopoietic progenitors as compared to BM cells. This is in line with a previous study that showed a higher expression of miR-196b in the Lin c-Kit^+^Sca-1^+^ compartment compared to the total BM cells; indicating that this miR-196b is necessary for the HSPCs maintenance and homeostasis (O’Connell et al., 2010). Previously, miR-196b has been reported as highly expressed in HSPCs and regulates cell survival and proliferation (Li et al., 2012). Moreover, miR-196b was reported to be abundant in mouse short-term hematopoietic stem cells (ST-HSCs) and undifferentiated CD34^+^ progenitors of which the expression was decreased following the onset of HSCs differentiation. Thus, this indicate the vital role of miR-196b in maintaining the undifferentiated state of primitive HSCs by repressing genes involved in differentiation (Popovic et al., 2009; Yekta, Shih & Bartel, 2004). The aberrant upregulation of miR-196b increased the proliferation and abnormal hematopoietic differentiation, which subsequently contribute to leukemogenesis as found in patients with MLL-associated leukemias (Popovic et al., 2009). Hence, 1,4-BQ may mediate leukemogenesis by upregulating the expression of miR-196b in HSPCs.

According to Wen et al. (2017), down-regulation of miR-196b suppresses proliferation of myelodysplasia syndromes-leukemia (MDS-L) cells, while upregulation resulted in increased cell proliferation. Our findings showed that 1,4-BQ upregulated MiR-196b starting at 2.5 µM indicating that 1,4-BQ has the potential to induce proliferative capacity in the myeloid and pre-B lymphoid progenitors. This is also in line with a previous study (Chow et al., 2018) that reported 1,4-BQ at non-cytotoxic concentrations caused aberrant expression of genes that are regulating self-renewing and differentiation of HSPCs, implying the vital role of lower dosage exposures to 1,4-BQ in mediating benzene hematotoxicity and leukemogenicity. Moreover, there is speculation that cells that survive apoptosis may acquire DNA damage (Dewi et al., 2019), hence may suggest that the cells that survived apoptosis upon 1,4-BQ exposure and acquired damage, tend to proliferate actively to produce LSCs.

In addition to miR-196b, this study also investigated the effect of 1,4-BQ exposure on miR-181a expression. MiR-181a has been proposed to play multiple roles in the progression of neoplasia and leukemia (Mori, Strano & Blandino, 2013; Shang et al., 2018; Taylor et al., 2013). However, inconsistency of the miR-181a expression in different hematological malignancies has been reported, indicating conflicting roles of these miRNAs that either act as tumor suppressors or onco-genes. For instance, miR-181a was found to act as an oncogene, with upregulation found in the pathogenesis of leukemia, though miR-181a was also found to be down-regulated in many other tumors and thus served as a tumor-suppressor gene (Bisso et al., 2013; Li et al., 2015; Shin et al., 2011). In previous studies of hematologic malignancies, miR-181a was found to be upregulated in acute myeloid leukemia (AML) (Debernardi et al., 2007) and myelodysplastic syndromes (Pons et al., 2009) but downregulated in multiple myeloma (Pichiorri et al., 2008) and chronic lymphocyte leukemia (Zhu et al., 2012). Therefore, as noted in the current study, the downregulation of miR-181a expression by 1,4-BQ at selected concentrations in BM cells, myeloid and erythroid progenitors reveal that this hematopoietic miRNA may act as a tumor suppressor gene and becomes a target for 1,4-BQ toxicity. This finding is also in agreement with a previous study conducted by Wei et al. (2015), which demonstrated downregulation of miR-181a in mice hematopoietic progenitors (Lin -c-Kit^+^) upon *in vivo* exposure to benzene.

On the contrary, a significant upregulation of miR-181a was observed in the pre-B lymphoid progenitors at the cytotoxic concentration (5 µM) of 1,4-BQ. Previously a study conducted by El-Khazragy et al. (2019) reported the upregulation of miR-181a in pediatric acute lymphoblastic leukemia (ALL) cases, indicating the role of miR-181a in pathogenesis of ALL. Similarly, a study by Lyu et al. (2017), also reported that ALL cell proliferation was enhanced following the ectopic expression of miR-181a. Thus, the reported evidence may explain the link of 1,4-BQ exposure to the pathogenesis of ALL by upregulating miR-181a expression specifically in the pre-B lymphoid progenitor as discovered in this current study. This is the first study that reported the association between an epigenetic factor and the pre-B lymphoid progenitor in mediating benzene toxicity as compared to previous studies concerning benzene toxicity that mainly focused on myeloid progenitors (Dewi et al., 2020; Zhang et al., 2012).

Next, we investigated the effect of 1,4-BQ exposure on miRNA-29a. In this study, 1,4-BQ caused significant downregulation in miRNA-29a expression in BM cells, myeloid and pre-B lymphoid progenitors upon 1,4-BQ exposure. MiR-29a is essential for HSCs self-renewing and is highly expressed in both human and mouse HSCs while being downregulated in differentiated progenitors (Han et al., 2010). A previous study by Han et al. (2010) reported downregulation in miR-29a expression in peripheral blood mononuclear cells obtained from AML patients. In addition, miR-29a expression has been reported to be downregulated in various tumors and acts as tumor suppressors (Garzon et al., 2008; Wang et al., 2012a). In addition, miR-29a plays an important role in promoting granulopoiesis and monopoiesis with decreased expression of miR-29a being responsible for acute myeloid leukemogenesis in AML patients (Wang et al., 2012a). They also highlighted the potency of benzene metabolites to cause damage in HSCs and progenitors *via* multiple mechanisms including epigenetic regulation that are responsible for initiation of LSCs population in benzene-induced hematological malignancies and BM depression. Moreover, miRNA-29a has been reported as a commonly found oncogene in human AML patients by promoting leukemogenesis which transforms normal myeloid progenitors to become LSCs (Han et al., 2010).

Collectively, the dysregulation of miRNAs as observed in this study upon 1,4-BQ exposure suggests that 1,4-BQ toxicity may promote the pathogenesis of hematological malignancies through self-renewing pathways regulating the HSPCs fate. Furthermore, our study demonstrated that the myeloid progenitor is the most affected in 1,4-BQ mediated toxicity as compared to BM, erythroid and pre-B lymphoid progenitors, suggesting that 1,4-BQ may mediate hematotoxicity and leukemogenicity targeting self-renewing and differentiation pathways of HSPCs *via* line-age-dependent mechanism. To date, this is the first study to uncover the role of hematopoietic miRNAs in mediating 1,4-BQ toxicity affecting the fate of HSPCs differential hematopoietic lineages, highlighting the need for more extensive studies to confirm this mechanism.

Transcription factors are known as the main regulator in controlling the HSPCs’ fate to undergo self-renewing and/or differentiation. Hence, involvement of transcription factors in benzene-induced toxicity targeting the HSPCs niche is worth exploring to construct a clear insight into the molecular mechanism of benzene-induced hematotoxicity/leukemogenicity. A previous study by Chow et al. (2018) has reported that 1,4-BQ caused significantly increased levels of HoxB4, Bmi-1 and GATA3 gene expression in exposed BM cells as compared to other self-renewing and differentiation genes such as GATA1, GATA2 and Wnt3. Furthermore, previous studies have also reported the involvement of transcription factors together with miRNAs in the mechanism of leukemogenesis due to their vital roles in the regulatory mechanism of hematopoiesis; mainly in the self-renewing and differentiation pathways (Kim, Civin & Kingsbury, 2019; Gocho & Yang, 2019; Mullany et al., 2018).

Our current finding demonstrated that 1,4-BQ exposure was able to induce upregulation in HoxB4, Bmi-1 and GATA3 proteins expression in a concentration dependent manner. Moreover, the HoxB4 expression was affected starting at the lowest 1,4-BQ concentration (1.25 µM) as compared to higher concentrations for Bmi-1 and GATA3. This indicates that the transcription factors controlling the HSPCs fate particularly the HoxB4 can be primarily targeted in mediating 1,4-BQ toxicity that is targeting the HSPCs niche. The HoxB4 gene is crucial for the maintenance of hematopoiesis by regulating the hemostasis between self-renewing and differentiation activities in HSPCs (Deng et al., 2013). A study by Umeda et al. (2012). has reported that BM cells samples in patients with AML showed significantly higher HoxB4 expression compared to negative controls, proving that HoxB4 also serves as an oncogene. Similarly, Bmi-1 was also reported to play an important role in the self-renewing activity of HSCs and cancer stem cells that can lead to the transformation of leukemic malignant cells (Sahasrabuddhe, 2016). Several previous studies have reported that higher expression of Bmi-1 was seen in a variety of human cancers including AML that emphasize the role of Bmi-1 in carcinogenesis (Sawa et al., 2005; Vonlanthen et al., 2001; Zhuang et al., 2008). Moreover, studies by Nishida et al. (2017) and (Sahasrabuddhe, 2016) showed that high Bmi-1 expression in AML patients is associated with poor prognosis and shorter survival in AML patients.

In addition to HoxB4 and Bmi-1, the increase in GATA3 protein as reported in this study is in line with a previous study by Chow et al. (2018) who showed that GATA3 gene expression is the most affected following 1,4-BQ exposure as compared to GATA1 and GATA2 genes. In fact, GATA3 is one of the transcription factors from the GATA family of which the expression is notable in several tissues including immune cells and its expression has been associated in the pathogenesis of ALL carcinogenesis (Crispino & Horwitz, 2017; Perez-Andreu et al., 2013). Furthermore, GATA3 protein is found to be highly expressed in fetal and adult thymus, lymphoma as well as in acute leukemia with T cell differentiation (Dorfman et al., 2017). In association to benzene exposure, a previous report by McHale et al. (2011) has highlighted that benzene exposure at low doses (less than 1 ppm) can significantly affect the T-cell receptor signaling pathway, indicating the involvement of the lymphoid lineage in benzene toxicity. Based on reports concerning the transcription factors involvement in the pathogenesis of hematological malignancies, our findings may suggest that the exposure to 1,4-BQ could promote hematological malignancies affecting differential hematopoietic lineages through dysregulation of self-renewing and differentiation pathways.

## Conclusion

In conclusion, 1,4-BQ induces aberration of miRNAs and transcription factors protein expression that are involved in regulating self-renewing and differentiation pathways of HSPCs. It was notable that 1,4-BQ exposure induced differential effects in mediating hematopoietic miRNAs expression between the BM cells in comparison to erythroid, myeloid and pre-B lymphoid progenitors. Moreover, the current study discovered that 1,4-BQ exposure was able to induce upregulation in HoxB4, Bmi-1 and GATA3 proteins expression in a concentration dependent manner. Additionally, the HoxB4 expression was found to be affected starting at the lowest 1,4-BQ concentration as compared to Bmi-1 and GATA3. This indicates that the transcription factors controlling the HSPCs fate, particularly the HoxB4, can be primarily targeted in mediating 1,4-BQ toxicity in targeting the HSPCs niche. Taken together, our finding lays a foundation for further mechanistic studies involving the roles of hematopoietic lineage, miRNAs and transcription factors in benzene-induced hematotoxicity and leukemogenicity that target the HSPCs niche.

##  Supplemental Information

10.7717/peerj.15608/supp-1 Supplemental Information 1 MicroRNAs gene expression

10.7717/peerj.15608/supp-2 Supplemental Information 2 Bmi-1 transcription factor protein expression

10.7717/peerj.15608/supp-3 Supplemental Information 3 GATA3 transcription factor protein expression

10.7717/peerj.15608/supp-4 Supplemental Information 4 Hox-B4 transcription factor protein expression

## Additional Information and Declarations

### Data Availability

The following information was supplied regarding data availability: The raw data for MicroRNA and transcription factors expression are available in the Supplemental Files.
